# Prebiotic Chemistry Experiments Using Microfluidic Devices

**DOI:** 10.3390/life12101665

**Published:** 2022-10-21

**Authors:** Karen Melissa Lerin-Morales, Luis F. Olguín, Eva Mateo-Martí, María Colín-García

**Affiliations:** 1 Posgrado en Ciencias de la Tierra, Universidad Nacional Autónoma de México, Ciudad de Mexico 04510, Mexico; 2 Laboratorio de Biofisicoquímica, Facultad de Química, Universidad Nacional Autónoma de México, Ciudad de Mexico 04510, Mexico; 3 Centro de Astrobiología (CAB), CSIC-INTA, Carretera de Ajalvir Km 4, Torrejón de Ardoz, 28850 Madrid, Spain; 4 Instituto de Geología, Universidad Nacional Autónoma de México, Ciudad de Mexico 04510, Mexico

**Keywords:** microfluidic devices, prebiotic chemistry, hydrothermal vents, mineral membranes, cellular compartmentalization

## Abstract

Microfluidic devices are small tools mostly consisting of one or more channels, with dimensions between one and hundreds of microns, where small volumes of fluids are manipulated. They have extensive use in the biomedical and chemical fields; however, in prebiotic chemistry, they only have been employed recently. In prebiotic chemistry, just three types of microfluidic devices have been used: the first ones are Y-form devices with laminar co-flow, used to study the precipitation of minerals in hydrothermal vents systems; the second ones are microdroplet devices that can form small droplets capable of mimic cellular compartmentalization; and the last ones are devices with microchambers that recreate the microenvironment inside rock pores under hydrothermal conditions. In this review, we summarized the experiments in the field of prebiotic chemistry that employed microfluidic devices. The main idea is to incentivize their use and discuss their potential to perform novel experiments that could contribute to unraveling some prebiotic chemistry questions.

## 1. Introduction

Microfluidic devices are, according to George Whitesides, “the science and technology of systems that process or manipulate small (10^−9^ to 10^−18^ L) amounts of fluids, using channels with dimensions of tens to hundreds of micrometers” [1]. It can also be said that microfluidics is the design and construction of small devices, including channels and chambers in microscale size, where the flow and mixing of fluids can be accurately controlled [1,2,3].

The multidisciplinary field of microfluidics emerged from a conjunction of technologies and principles of physics, biology, microtechnology, material science, chemistry, and microelectronics, among others [4,5]. Precedents to microfluidics were microanalytical methods, such as gas phase chromatography (GPC), high-pressure liquid chromatography (HPLC), and capillary electrophoresis (CE), all in the capillary format; these techniques entirely changed chemical analysis [1].

Microfluidic devices are used in biological [6,7,8,9,10] and chemical essays [11,12], clinical and forensics [13,14,15], molecular and medical diagnosis [16,17,18,19], pollution monitoring [20,21,22], drug design and therapeutic activity [23,24,25,26], and biohazard detection [7,27,28,29], among others disciplines.

There are multiple advantages to using microfluid devices. The most evident is that miniaturization reduces reagent consumption, thus cost and waste are diminished too. Other advantages are high precision of the mixing regimes and control of fluids [4], as well as the possibility to manipulate multiphase flows (i.e., liquid, gases, particles) [1]. Diffusive mixing is fast and increases the speed of reactions. In microfluidics devices, sample processing is fast [17], and it is possible to work under supercritical conditions at the microscale [11]. The dynamics of interfaces in complex systems can also be explored [30]. Compared to macroscopic systems, there is an improvement in thermal and mass transfer due to the high surface-to-volume ratios on these devices [31]. Microfluidic devices enable quick temperature adjustments and accurate temperature control. The concentration of the molecules both in space and time is feasible [1]. Additionally, the huge design flexibility of these devices is remarkable [32]. In a single-designed integrated device, such as lab-on-chip (LOC), a full experiment could be performed, including sampling, processing, and measurements, thanks to the combination of microfluidic channels and active or passive components [33].

### 1.1. Components

A microfluidic system consists of different components: (i) a device for introducing samples and reactants; (ii) a method for pushing, mixing, and combining the fluids; and (iii) other components (detectors, purification tools, etc.) [1].

- Accessories for introducing samples. Samples can be loaded in different ways: (i) manually, (ii) by integrated capillaries that transfer the fluid directly by vacuum, (iii) by capillaries connected via clamping or screwing; or (iv) by dispensing systems activated by short pressure pulses [34].
- Methods for pushing, mixing, and combining fluids. The dispositive needs components, either active or passive structures, arranged in such a manner that guide liquids through channels, channel networks, or chambers. Depending on the nature of the experiments, some adaptations can be made, including (but not limited to) microvalves installation (for blocking/unblocking channels), pumps (for promoting/increasing fluid flow), and micromixers [34]; micromixers can be active (requiring external activation) and passive mixers [35].
- Other components. All needed components can be added for processing or analyzing the samples. For example, samples can be sorted according to their size for filtration; in classic filtration, components transported on the flow are retained [34]. Filtration can also be accomplished in membranes [36] or by centrifugal forces in centrifugal platforms [37]. The inclusion of solid-phase chromatography extraction elements is also applied [38]. In microfluidic devices, sometimes it is necessary to measure and control the temperature [39].

### 1.2. Materials for Microfluidic Devices Construction

Microfluidic devices can be fabricated from different materials and following different techniques; there is continuous design and improvement in this regard [5]. The material is critical because it affects the flow, biocompatibility, absorptivity, and function of the components [40]. Some materials that have been used comprise thermoplastic polymers (polycarbonate), elastomers (polydimethylsiloxane), hydrogels, inorganic substrates (glass, silicon), paper, resins, ceramics, or a combination of these materials [41]. For more detailed information, consult the work of Niculescu [5].

### 1.3. Basic Principles of Microfluidics

The manipulated volume of fluid in microfluidic devices is not so relevant. The most significant feature of these devices is the length scale in the channels that allows laminar flow of the fluid [42]. In laminar flow, the fluid moves smoothly as if layers slip over each other [43]. The Reynolds number (*Re*) is a dimensionless number that shows the flow regime as a function of the density (ρ) and viscosity (µ) of the flow, the flow velocity (ν), and the duct diameter (D) where the fluid flows. The Reynolds number can be expressed as follows:

(1) $$Re=\frac{\nu D\rho }{µ}$$

Flows with high Reynolds numbers tend to be turbulent, whereas flows with low Reynolds numbers are laminar. For practical applications of flows in ducts, if the Reynolds number is below 2000, the flow regime is laminar; if the Reynolds number is above 4000, it is turbulent. Regimes at intermediate values of Reynolds numbers between 2000 and 4000 are named transition regimes [44]. In microfluidic devices, the Reynolds number is usually smaller, with values less than 1 [45].

### 1.4. Types of Microfluidic Devices Configurations

**Figure 1:**
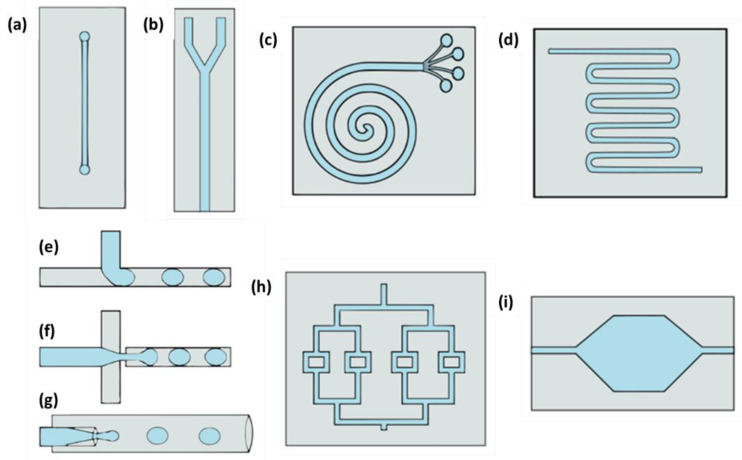
Types of microfluidic devices channel configurations: (**a**) straight, (**b**)”Y” shaped, (**c**) spiral, (**d**) serpentine, (**e**) “T”-junction, (**f**) cross-junction, (**g**) flow-focusing, (**h**) division, and (**i**) microchamber.

In microfluidic devices, the channel design will depend on the device’s function, and devices can come up with various types of channels. Some types of channels are straight, Y-form, T-junction, spiral, cross-junction, flow-focusing, division, serpentine, and microchambers (Figure 1). Devices with spiral and Y channels are commonly used for separations (although Y channels are used for combining fluids as well). To carry out the mixing of fluids, a serpentine design is employed, and the division channels are used for splitting fluids. T-junction, cross-junctions, and flow-focusing are commonly used in microdroplet devices, and in microchambers, physical, chemical, and biological reactions are performed.

The necessity to reproduce conditions occurring in some environments has led researchers to conceive experiments that permit the integration of various factors and/or simplify the way such experiments are carried out. Microfluidic devices facilitate the reproduction of certain prebiotic environments, and for that reason, they have been used to perform prebiotic chemistry experiments. Three types of microfluidic devices have been used for this purpose: Y-form devices with laminar co-flow, microdroplet devices, and devices with microchambers. The first is commonly employed to study mineral precipitation, under conditions simulating those of hydrothermal systems; the second type is used to imitate cell compartmentalization; and the last is used to recreate rock pores in hydrothermal environments. In some cases, the type of device does not match the mentioned purpose. In this review, the prebiotic chemistry experiments performed with microfluidic devices are summarized and their use to accomplish novel experiments that contribute to the development of this field is analyzed.

## 2. Microfluidic Devices and Prebiotic Chemistry

### 2.1. Mineral Precipitation and Mineral Membranes on Hydrothermal Systems

Since its discovery in the late seventies [46], submarine hydrothermal vent systems have been proposed as environments where life could have emerged [47,48,49,50,51,52]. This idea arose after considering the physical and chemical gradients of hydrothermal vents as energy sources for the abiotic synthesis of organic molecules [47,53,54,55]. There are two types of submarine hydrothermal systems designated as prebiotic environments: black and white smokers. Each one has certain attributes, but they can coexist in the same hydrothermal field [56,57].

Black smokers are systems found near oceanic ridges, and they form when oceanic waters seep into the oceanic crust and are heated by magma [57]. As the oceanic water heats up, they begin to return to the ocean, and due to elevated temperatures (400 °C) and acidic pH, metals are dissolved from the crust rocks and carried with water [57]. When the water comes up from the crust and contacts the low-temperature ocean, sulfides precipitate, and they are the main responsible for the black smoke appearance [56,58]. On the other hand, white smokers are more recently discovered hydrothermal systems found farther away from the heat source than black smokers. White smokers’ temperature is rather warm (40–90 °C), their pH is alkaline (9–11), and within them are mainly carbonate precipitates [59,60]. Due to the fewer extreme characteristics of white smokers, they are pointed out as favorable places for the beginning of life [55,61,62].

In the study of hydrothermal vents systems (HVS) as prebiotic environments, laboratory simulations, to recreate the conditions in these systems, have been scarcely employed. These experiments must include factors such as pH, pressure, temperature, and the use of minerals identified in these types of environments [63]. One way to simulate the reactions on HVS is by bringing two fluids in contact; one mimics hydrothermal fluids, and the other simulates oceanic water, just as it happens in hydrothermal systems. The solution simulating the hydrothermal fluids is alkaline, and the solution simulating oceanic water is acidic. Both solutions may contain soluble metal salts and sodium silicate compounds [64,65,66,67].

Different methods can be employed to put the two solutions in contact, e.g., by adding drops of one solution to the other [54,68] or by injecting the hydrothermal fluid into a reservoir containing the oceanic solution [64,65,66,67]. The contact of the two solutions creates hollow structures called chemical gardens [69,70]. Chemical gardens are thus inorganic structures formed by the reaction of a soluble metallic salt and an aqueous solution of anions [71].

**Figure 2:**
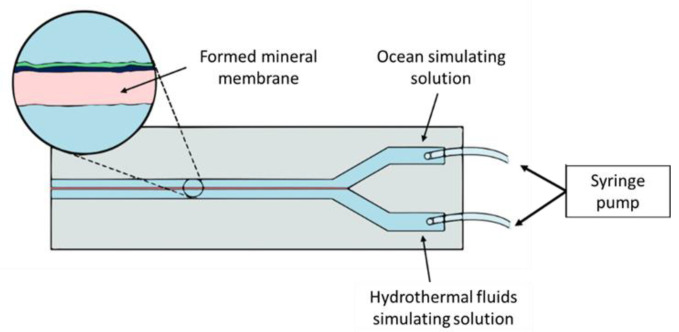
“Y”-shaped channel microfluidic devices design for simulating mineral precipitation in hydrothermal systems.

As an approach to studying chemical gardens more easily, Batista and Steinbock [72] created microfluidic devices for mineral membrane precipitation. Such devices are fabricated with low-cost materials such as acrylic, Parafilm^®^, polystyrene, glass, or Teflon^®^. Fabrication of microfluidic devices for mineral precipitation is simple; for example, a cut Y-pattern parafilm is sandwiched between two acrylic sheets with drilled inlets. Barb fittings are glued to the perforations to connect the tubing [72]. The Y-shaped channel configuration (Figure 2) permits the flux of two parallel streams without mixing. Although the streams do not mix, they are in contact with each other, allowing the creation of conditions far from equilibrium (pH, temperature, and concentration gradients) [45]. For instance, Möller et al. [73] prove the existence of pH gradients up to six units at micrometric scales in the microfluidic device channel.

In many of the experiments of mineral membrane precipitation, as in chemical gardens, two reactants are used to simulate the ocean and the hydrothermal fluids. One is an acidic solution that contains metals, and the other is a NaOH alkaline solution (Table 1). Different compositions of solutions with different metals in the form of chlorides have been used. Some examples are chlorides of Mg^2+^, Mn^2+^, Fe^2+/3+^, Co^2+^, and Cu^2+^ [72]. When the two solutions come into contact, in the interface, a mineral membrane is formed by precipitation. Its composition depends on the metal salt employed. In experiments with cobalt salt, Co(OH)_2_ and cobalt oxide are produced [74], while membranes formed with manganese chloride are composed of Mn(OH)_2_ [72]. Further, when membranes were produced in the presence of iron chloride and phosphate, the composition of the membrane consisted of the goethite and vivianite minerals [75].

Some mineral membrane characteristics have been studied. For example, Wang and Steinbock [76] explore the effect of temperature in the formation of Ni(OH)_2_ membranes, finding that the membrane growth rate is independent of the temperature in the interval of 10 to 40 °C. In another experiment, Ding et al. [77] study mineral membranes formed by Mn(OH)_2_ and observe the formation of waves on the membrane surface. Such waves facilitate ion transport in the membrane; thus, they may be relevant in studying membrane transport in protocells.

The catalytic properties of these membranes have been also explored. Wang et al. [75], using membranes formed at different pH gradients and with different metal salts, explored if the membranes could synthesize pyrophosphate from acyl-phosphate. They found that membranes formed under high pH gradients and containing Fe^2+^ favored the yield of pyrophosphate. In two independent experiments, Sojo et al. [78] and Vasiliadou et al. [79] probed the capacity of (Fe, Ni)S membranes to reduce CO_2_ in the presence of H_2_, obtaining negative results, concluding that step pH gradients are not enough to reduce CO_2_. They suggested that the reaction could be achieved at pressures greater than atmospheric. In a further experiment by Hudson et al., using the same factors as Sojo et al. [78], except for the H_2_ pressure (1.5 bar in this study), the CO_2_ reduction to formate, promoted by a pH gradient, was observed [80].

Mineral membrane precipitation experiments in microfluidic devices with Y-shaped channels are recent and have the potential to be used in the fields of prebiotic chemistry and material synthesis [45]. The exploration of various experimental conditions, such as different reactants and their concentration, and different physical and chemical conditions, such as temperature or pH, and the presence of prebiotic molecules is still missing. An important matter is to evaluate the potential of mineral membranes as catalysts and concentrators of relevant organic molecules in the prebiotic chemistry field. Additionally, this kind of microfluidic device has traits that allow the generation of physical and chemical gradients. Those gradients can be useful in other types of experiments necessary to examine non-equilibrium processes, which are important in prebiotic environments.

**Table 1:** Prebiotic chemistry experiments in microfluidic devices with Y-shaped channels.

Experiment Type	Device Description	Experimental	Findings	Reference
Mineral precipitation in hydrothermal systems	Parallel laminar flow, Y-shaped channel, external temperature control.	Ni(OH)_2_ mineral membranes form from NaOH and NiCl_2_ solutions, at different temperatures.	From T-10 to 40 °C, the effective diffusion coefficient is temperature independent.	[76]
Mineral precipitation in hydrothermal systems	Parallel laminar flow, Y-shaped channel.	Membrane precipitation by alkaline inorganic phosphorous, acyl-phosphate solution, and acidic solution including cations (Fe^2+^, Fe^3+^, Ca^2+^, Mn^2+^, Co^2+^, Cu^2+^, Zn^2+,^ or Ni^2+^). Precipitated membrane was incubated in a water bath for 1 h at 38 °C and analyzed.	Fe^2+^, other divalent cations and Fe^3+^ promote the formation of pyrophosphate from inorganic phosphorus and acyl-phosphate.	[75]
Mineral precipitation in hydrothermal systems	Parallel laminar flow, Y-shaped channel, heated by a heating plate.	Membranes formed from alkaline (Na_2_S, Na_6_Si_2_O_7_, pH 11) and acidic (FeCl_2_, NiCl_2,_ and NaHCO_3_, pH 6) solutionsH_2_ were introduced into the alkaline solution.	Fe(Ni)S mineral membrane and a pH gradient of 5 units formed. The reaction between H_2_ and CO_2_ at the mineral was not possible at atmospheric pressure.	[79]
Mineral precipitation in hydrothermal systems	Parallel laminar flow, Y-shaped channel, heated by a plate.	Membranes formed by alkaline (pH 11, Na_2_S, K_2_HPO_4_, Na_2_MoO_4_, H_2_ at atmospheric pressure) and acidic (pH 6, FeCl_2_, NiCl_2,_ and CO_2_ at atmospheric pressure) solutions. The reaction times were 0, 0.5,1, 2, 5, 12 and 24 h.	CO_2_ reduction was not achieved in the presence of the Fe(Ni)S membrane. High pressures of H_2_ are required to achieve CO_2_ reduction.	[78]
Mineral precipitation in hydrothermal systems	Parallel laminar flow, Y-shaped channel, microfluidic pumps driven by pressure.	n alkaline (pH 12.3, Na_2_S, Na_2_Si_3_O_7_, degassed water, 1.5 bar of H_2_) and acidic (pH 3.9, FeCl_2_, NiCl_2_, 1.5 bar of CO_2_) solutions.	CO_2_ reduced to formate in a Fe(Ni)S mineral membrane promoted by a pH gradient. H_2_ oxidation, and movement of electrons across the mineral membrane to reduceCO_2_.	[80]
Mineral precipitation in hydrothermal systems	Parallel laminar flow, Y-shaped channel.	Membranes from NaOH and MnCl_2_ solutions at different concentrations. A theoretical model of electron transport was made.	The waviness enhances the diffusion across the Mn(OH)_2_ membrane.	[77]
Pores in rocks in hydrothermal systems	Parallel laminar flow, Y-shaped channel, platinum electrodes for electric potential measurement.	Na_2_S at pH 11.8 and a FeCl_2_ at pH 5.8 solutions.	A chemical gradient, 6 pH units of difference, at a micrometric scale. Precipitation reaction stabilizes the pH gradient and makes it larger.	[73]

### 2.2. Prebiotic Chemistry Experiments with Droplet-Based Microfluidic Devices

There are three minimum characteristics for a system to be considered alive: (i) metabolism, which consists of a reactions network capable of synthesizing useful molecules; (ii) self-replication, which refers to a system capable of producing macromolecules with a template, by polycondensation of molecules generated by metabolism; and (iii) membranes, structures that delimitate and protect the living system from the exterior; the membrane must be permeable and have the capacity to grow using the molecules produced by metabolism [81,82]. It is believed that membranes were the first protobiological structures on early Earth [83]. Cellular life probably emerged when self-assembling membranes captured in their interior catalytic and informational polymers [83]. Moreover, compartmentalization is essential for the emergence of Darwinian evolution since, in this way, different catalytic/informational systems are well delimited one for another [84].

**Figure 3:**
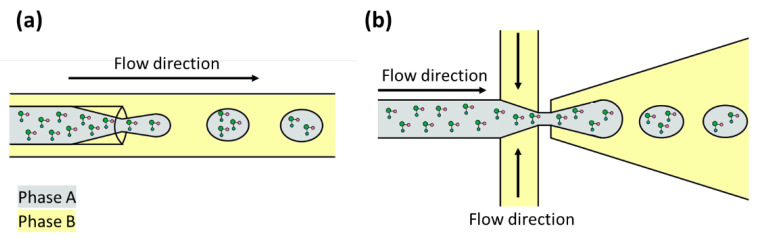
Encapsulation of molecules with droplet-based microfluidic devices. Phases A and B are immiscible liquids. (**a**) Flow-focusing geometry. (**b**) Cross-junction geometry.

In prebiotic chemistry, various methods of compartmentalization have been considered. The principal is encapsulation by vesicles made of amphiphilic molecules. Amphiphilic molecules possess polar (hydrophilic) and non-polar (hydrophobic) moieties. The polar component consists of hydroxyl, carboxyl, amine, phosphate, or sulfate groups, while the non-polar part comprises a hydrophobic hydrocarbon chain [83]. At specific conditions of temperature, pH, salt concentration, or biopolymer presence, the amphiphilic molecules self-assemble into vesicles with an aqueous interior separated from the medium by one or two bilayers [85]. The self-assembly process of amphiphilic molecules to vesicles has been studied under various conditions, including the interaction with molecules that can be encapsulated such as amino acids, sugars, nitrogen bases, and even with macromolecules as peptides [86]. Droplet-based microfluidic devices are an alternative to studying the compartmentalization phenomenon since they have been used to perform confined chemical and biochemical reactions [87,88,89]. These devices have geometries that allow them to intersect two non-miscible fluids, and thanks to this intersection, monodisperse microdroplets are created (Figure 3) [87,90]. Microdroplets are discrete units with specific microenvironments that favor reactions that probably occurred in early Earth compartments [91]. There are few studies describing the use of droplet-based microfluidic devices to address prebiotic chemistry problems and their principal objective is to investigate processes that can lead to Darwinian evolution (Table 2). The studies are described below.

Doran et al. [92] designed a microfluidic device consisting of a droplet generator, an incubation microchamber, and a droplet size sorter. In the study, the authors assume that larger droplets with high osmolarity are the most suitable to suffer evolutive processes. So, in the device, droplets with catalytic networks would be created, then incubated, and those with the highest polymer concentration (i.e., the biggest) would be selected for another incubation cycle. To test the device two kinds of droplets were placed in the incubation chamber: one with water and the other with glycyl-glycine. The results show that glycyl-glycine droplets grew at the expense of water droplets.

In a different experiment, Ameta et al. [93] used a device to create microdroplets loaded with RNA networks that catalyze their own formation. They analyzed the accumulation of products (growth) and the fraction of the networks’ catalytic species (composition) as a function of their topology. Furthermore, the authors studied the reproduction (interpreted as species accumulation) and the variation (changes in the fraction of species). As a result, they found that strong variations surge from the new catalytic species that perturb networks with weak connections and that growth increases with the global connectivity of networks.

There is another way to create droplets without amphiphilic molecules or non-miscible fluids. The use of solutions of two polyelectrolytes, with opposed charges, leads to spontaneous liquid–liquid phase separation and the formation of droplets called complex coacervates [94]. This kind of coacervates can be created by microfluidic devices, as shown by van Swaay et al. [95]. Complex coacervates were made from poly diallyl dimethylammonium chloride (PDDA) and ATP or PDDA and carboxymethyl-dextran (CM-dextran). In the experiment, DNA oligonucleotides were added to the coacervates flow. The aim was to detect if the genetic information (DNA oligonucleotides) could be immobilized in the coacervates or be transferred. The authors showed that two populations of coacervates, with different DNA oligonucleotides, can coexist for up to 48 h without information exchange.

The synthesis of prebiotic molecules can be achieved in microdroplets. A study by Ju et al. [96] shows that microdroplets favor the phosphorylation of adenosine, guanosine, uridine, and cytidine in the presence of KH_2_PO_4_ (as a phosphate source), at ambient conditions and without a catalyst. Normally, this synthesis cannot be achieved in bulk aqueous solution, but the formation of microdroplets gave a negative ∆G, allowing the synthesis to take place. Further, under the same conditions in microdroplets, the polymerization of nucleotides in dimers is possible.

In the studies presented here, the microdroplets do not have properly a membrane, although it is possible to create it in microfluidic devices. Many studies are focused on the formation of liposomes, including encapsulated molecules with pharmaceutical applications [97]. Majumder et al. [89] have described droplet microfluidic methods to create membranes for the creation of artificial cells for the biomedical field. The same method can be applied to fabricate prebiotic membranes. For example, it would be interesting to include amphiphilic molecules with prebiotic relevance. Another possibility is the simultaneous use of amphiphilic molecules and another variety of prebiotic molecules that can be encapsulated (e.g., amino acids, nitrogenous bases; carboxylic acids, sugar precursors, or their polymers). In this way, it would be possible to analyze the occurrence of synergies between organic molecules that make up protocells. These synergies are important for protocell formation, protometabolism, replication, and evolution of these structures. By employing microfluidic devices, various systems with different combinations of molecules can be tested [86]. At the same time, microdroplets offer the potential to analyze reaction processes in confined areas, including microenvironments and different conditions, both from each other and from the outside medium as demonstrated by Ameta et al. [93]. These processes are important in prebiotic chemistry since they can give rise to Darwinian evolution.

Compartmentalization is a feature of living systems, and some authors suggest that it is important for the concentration and/or protection of organic molecules synthesized in prebiotic environments. Experimentally, compartmentalization can be spawned by fabricating microdroplets with encapsulated molecules. There are several methods to prepare them without the use of microdroplet devices. These methods already have extensive use not only in prebiotic chemistry but also in other fields and are older than microdroplet devices. This is a probable reason why compartmentalization in a prebiotic chemistry context is generally studied by using “traditional methods”. Droplet microfluidics usage is being explored recently by only a few groups. However, there are some advantages of microfluidic devices over traditional methods, such as: (i) a massive production of monodisperse microdroplets, (ii) the precise generation and repeatability of droplets operation, and (iii) the possibility of encapsulating molecules into the droplets and use them as microreactors [87,88,98].

**Table 2:** Prebiotic chemistry experiments in droplet-based microfluidic devices.

Experiment Type	Device Description	Experimental	Findings	Reference
Cellular compartmentalization models	Double droplet generator, flow-focusing channel arrangement, microchamber, droplet size sorting (pressurized air), droplet spitter, and fuser.	Water and glycyl-glycine droplets created and incubated in the microchamber.	Osmotic exchange between water and glycyl-glycine droplets. Glycyl-glycine grows at expense of water droplets.	[92]
Cellular compartmentalization models	Flow-focusing geometry.	DNA oligonucleotides labeled (fluorescein or cyanine) added to a flow of coacervates (poly(dialyl dimethylammon ium chloride)), ATP or carboxymethyl-dextran].	Two populations of DNA oligonucleotides coexist near each other without genetic information exchange for up to 48 h.	[95]
Reaction networks evolution	Four devices: (i) “T” junction, (ii) droplet fusion by electrocoalescence and incubation, (iii) droplet split, and (iv) droplet fusion by electrocoalescence and incubation.	Microdroplets with catalytic RNAs fragments and hairpin RNA reporters, incubated (48 °C/1 h) and split. Labeled with barcoded DNA and incubated (60°/1 h). RNA fraction of each droplet measured by next-generation DNA barcoded sequencing.	The final fraction of RNA species depends on the composition of the network. Networks with greater yield show fewer perturbations.	[93]
Prebiotic synthesis	Two devices: (i) sparyer similar to ESSI ^a^, connected MS ^b^, (ii) cylindrical chamber, ceramic atomizer, heating tape.	Nebulization of a solution (adenosine, guanosine, uridine, cytidine, KH_2_PO_4_) with the two devices.	Produced microdroplets have negative ∆G, allowing ribonucleosides phosphorylation and polymerization under ambient conditions.	[96]

^a^ Electrosonic spray ionization, ^b^ mass spectrometry.

### 2.3. Prebiotic Chemistry Experiments in Microfluidic Devices with Microchambers

Proposed terrestrial environments for life appearance are diverse, from the primitive ocean, hydrothermal vents (submarine and subaerial), subaerial exposures, water bodies, and oceanic ice [99]. Each environment favors particular and relevant mechanisms for prebiotic chemistry but disfavors others. Nevertheless, the presence of microenvironments is key for prebiotic reactions, since they exhibit unique traits that make them different from the whole environment.

Stüeken et al. [99] highlight the possible microenvironments in the early Earth and how these environments could have contributed to the synthesis and accumulation of organic compounds. They propose that each microenvironment has attributes that allow processes different from the others. In addition, the authors say that life could have required the contribution of all those microenvironments to arise. This idea implies that global transport was needed to communicate the microenvironments and interchange the feedstock for the emergence of life.

**Figure 4:**
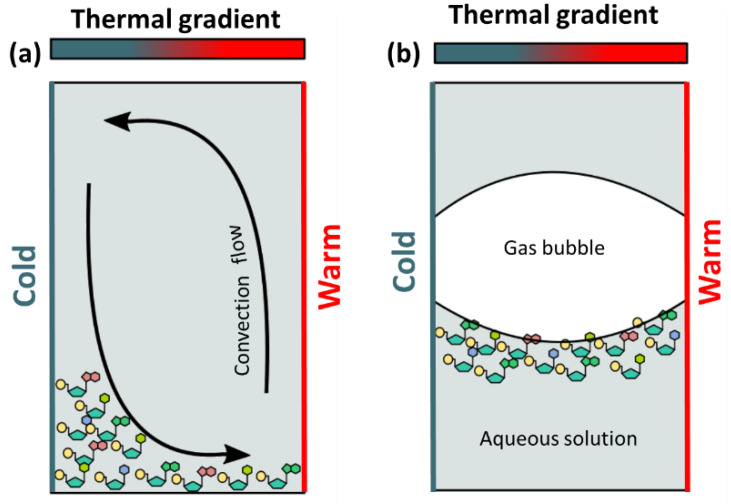
Accumulation of molecules in thermal traps: (**a**) thermal trap filled with an aqueous solution: molecules accumulated in the bottom of the capillary (by gravitational forces) and on the cold side (by convection induced by the thermal gradient); (**b**) thermal trap filled with an aqueous solution and gas: molecules tend to accumulate at the gas–water interface.

Microfluidic devices with microchambers (millimeter size) represent an approach to model processes that could occur in microenvironments. Dieter Braun’s research group is using these devices to study the microscale effects that may have occurred in pore rocks at hydrothermal environments. It is worth mentioning the series of papers in which the effects of thermophoresis (particle movement driven by thermal gradients) on DNA are analyzed. To study this phenomenon the authors constructed devices called “thermal traps” (TT), which are made of various materials and allow the creation of thermal gradients (Table 3). The thermal gradient is created by: (i) heating a capillary with an IR laser [100]; (ii) heating one side and cooling the other side of the capillary [101], or (iii) heating the two sides of a microchamber at different temperatures [102]. In TTs, convection currents are generated in which the flow is upward on the hot side and downward on the cold side of the chamber. If dissolved molecules are present in a thermal trap, they are pushed toward the cold side and accumulate at the bottom of the chamber by the effects of both thermophoresis and gravity (Figure 4). This effect is called thermogravitational accumulation [103]. If the chamber contains gas besides the solution of molecules, the molecules accumulate in the gas-water interface (Figure 4) [104]. By placing DNA molecules in solution in TTs, Braun’s group was able to observe their accumulation [100], replication [105], elongation [106] and the replication and subsequent selection of long DNA chains over short [101].

In more recent works, Braun’s group introduced the existence of interfaces and other molecules, besides DNA, in their experiments. For example, Ianeselli et al. [107] employed a Teflon microchamber as a TT, loaded it with gas and water, and then applied heat. This induced the formation of a microscale analog of the water cycle and the consequent formation of gas–liquid interfaces in the microchamber. The authors performed further experiments with a DNA–saline buffer solution instead of pure water and observed fluctuations in the salt concentration in the gas–water interface that promote DNA string division below its melting point temperature. Further experiments showed that DNA replication was possible in the microenvironments created in the devices. Ianeselli et al. [108] observed the creation of two ideal settings for DNA replication in a microfluidic chamber: one favorable for the denaturation of DNA or RNA (droplets with lower pH and salt concentration) and the other with characteristics for replication (higher salt concentration and neutral pH). On the other hand, Morasch et al. [104] used a corrugated microchamber as a TT to create air–water interfaces. In these experiments, besides DNA, they included other molecules such as RNA, ribose aminooxazoline, and cytidine nucleotides. Various relevant mechanisms in the prebiotic context were observed: an increase in RNA catalytic activity in the gas–water interface, DNA and RNA accumulation up to the formation of hydrogels, ribose aminooxazoline crystallization, and phosphorylation of cytidine nucleotides.

In addition to DNA and RNA molecules, Braun’s group has also studied larger structures such as vesicles and coacervates in thermal gradients. Morasch et al. [104] introduced oleic acid vesicles in a microchamber filled with a DNA solution and gas. They noted the vesicle aggrupation in clusters and the DNA encapsulation inside the vesicles when applying a thermal gradient. In another experiment, Ianeselli et al. [102] explored the behavior of coacervates under a thermal gradient. Coacervates tend to accumulate and grow by fusion on the interface gas–water and the forces in the chamber (heat and gas bubbles movement) promote the coacervate segregation in two different populations: oligonucleotide-polypeptide coacervates, in the aqueous bulk; and sugar-oligonucleotide-polypeptide coacervates, in the gas–water interface.

Besides studying thermal gradients, microfluidic devices have been used to approach the chirality bias in biomolecules in a novel way. Sun et al. [109] used a microfluidic device, consisting of ten pairs of inclined chambers, to imitate rock micropores in hydrothermal systems, within which microvortices can form. The authors concluded that laminar microvortices could induce enantioselectivity in supramolecular systems composed of non-chiral molecules.

Compartmentalization was also explored using microfluidic devices with microchambers. In a device composed of serial microchambers and microchannels, Sugiyama et al. [110] caught and selected liposomes by size. Afterwards, the liposomes were exposed to a uranine and fructose solution, resulting in the encapsulation of the molecules in the liposomes, even against the concentration gradient. The authors argue that this mechanism can provide clues in understanding the continuous development of protocells in early Earth.

The experiments performed by Braun’s group are relevant because they highlight the importance of prebiotic microenvironments, where processes not observed at large scales can occur. All the works of this research group are within the RNA world theory framework, nevertheless, it would be interesting to perform experiments with other types of molecules such as amino acids, carboxylic acids, sugars, or their precursors. This will allow us to observe their behavior in microenvironments and to test if it is possible to observe processes, such as concentration, polymerization, or catalysis. On the other hand, the experiments performed by Sun et al. [109] and Sugiyama et al. [110] show us how microfluidic with microchamber devices also can be used in novel ways to approach various issues related to prebiotic chemistry such as chirality and compartmentalization.

**Table 3:** Prebiotic chemistry experiments in microfluidic devices with microchambers.

Experiment Type	Device Description	Experimental	Findings	Reference
Simulation of Pores in rocks in hydrothermal systems	Thermal trap. A microchamber heated by an infrared laser.	Fluorescent-stained DNA heated in the capillary, under a temperature gradient.	DNA thermal diffusion coefficient was measured. DNA accumulates in the lower part of the chamber, near the heating spot (from nmol/L to µmol/L).	[100]
Pores in rocks in hydrothermal systems	Thermal trap. Borosilicate capillary embedded in immersion oil and inserted between a silicon plate and a sapphire cover. An infrared laser as heat source.	PCR ^a^ solution and DNA oligonucleotide templates (random sequences) stained with fluorescent dye heated by a temperature difference of 27 K.	Temperature gradients trigger replication and accumulation of short DNA by thermophoresis and convection.	[105]
Pores in rocks in hydrothermal systems	Thermal trap. Borosilicate capillary embedded in immersion oil, inserted between silicon plate and sapphire cover. Infrared laser as heat source.	Double-chain DNA segments capable of reversible union by hybridization heated.	Thermal gradient promotes DNA accumulation and polymerization	[106]
Pores in rocks in hydrothermal systems	Thermal trap. Borosilicate capillary inserted between two metallic plates, temperature controlled heated on one side and cooling the other side.	DNA, Taq polymerase, fluorescent dye heated with temperature gradients (38 °C to 71 °C). DNA in PCR buffer (6 µm/s flow) heated with gradients (36–73 °C; 61–94 °C).	Temperature gradients promote replication of DNA oligonucleotides with a sequence length. Long-over short sequences are preferred.	[101]
Pores in rocks in hydrothermal systems	Three inlets, two outlets, ten pairs of asymmetric inclined microchambers, allowing microvortice formation.	BTAC ^b^ and DMF ^c^ introduced (1 mL/h) in the central inlet. DMF/H_2_O is introduced (30 mL/h; 40 °C) at the side inlets. TPPS_4_^d^, H_2_SO_4_, and H_2_O injected (30 mL/h) in the side inlets, and in central inlet, a C_2_mim^+ e^ and HCl dissolution (1 mL/h).	Chiral microvortices created in the microchambers induce hydrodynamic selection of enantiomers in supramolecular systems composed of non-chiral molecules.	[109]
Pores in rocks in hydrothermal systems	Thermal trap. Microchamber made of Teflon and placed between a sapphire plate (heated) and a silicon plate (cooled).	Microchamber filled with air and dissolution of DNA chains (labeled with a chromophore) on a salt buffer (EDTA and NaCl), a temperature gradient from 9 °C to 15 °C was applied.	Formation of a mini water cycle analog that induces fluctuations in salt concentrations in the air–water interface promoting periodic separation of DNA strands below their melting temperature.	[107]
Pores in rocks in hydrothermal systems	Thermal traps. Corrugated microchambers (PETG plastic, UV-curable resin, or Teflon) sandwiched between sapphire (heated) and silicon (cooled) plates.	Temperature gradients applied. Devices filled with gas and solutions of DNA, RNA, ribozymes, ribose aminooxazoline, cytidine nucleosides, and monoammonium phosphate or vesicles (oleic acid or 1,2-Dioleoyl-sn-glycero-3-phosphocholine and oligonucleotides).	DNA and RNA form hydrogels and ribozymes, increase catalytic activity at the gas–water interface. Nucleotide encapsulation in vesicles, ribose aminooxazoline crystallization, and cytidine nucleosides phosphorylation.	[104]
Cellular compartmentalization	Arrangement of serial channels and microchambers.	Liposomes of phospholipids, cholesterol, and fluorescent dye doped with fructose trapped in the microchambers, exposed to a uranine/fructose and fluorescein-12-adenosine triphosphate solution (ATP analog) different flows and pH.	Fructose, uranine, and ATP analog accumulation in the liposomes, even against concentration gradient (between liposome and exterior).	[110]
Pores in rocks in hydrothermal systems	Thermal traps. Triangular PTFE plastic sheets placed between sapphire (heated) and silicon (cooled) plates.	Different coacervates compositions (CM-Dex ^f^ or ATP, with pLys ^g^ or PDDA ^h^ in Na^++^bicine or tris buffer), temperature gradient, gas volume, and microchamber thickness. Some experiments included RNA.	The fusion, accumulation, and division of coacervates occurred at the gas–water interface. Two coacervate populations can be separated: in the gas–water coacervates of RNA, CM-Dex, and pLys; in the bulk, coacervates of RNA and pLys.	[102]
Pores in rocks in hydrothermal systems	Thermal trap. Cut Teflon sheet placed between a silicon plate (covered with Teflon) and a sapphire plate. Thermal gradient by differential heating of plates.	Microchamber was filled with CO_2_ at different pressures and solutions Lysosensor Yellow/blue dye, RNA, MgCl_2,_ and buffer Tris; and DNA nucleotides, Taq polymerase, complementary primers, MgCl_2_, Tris Buffer, KCl, and (NH_4_)_2_SO_4_. Temperature gradients (5 °C to 17 °C).	Dew cycle generation. In dewRNA or DNA, melting is favored, in the bulk solution. Emergence of larger DNA strands was observed.	[108]

^a^ Polymerase chain reaction, ^b^ tris(ethyl cinnamate) benzene-1,3,5-tricarboxamide, ^c^ N,N-dimethylformamide, ^d^ tetra-(4-sulfonatophenyl) porphyrin, ^e^ 1-ethyl-3-methylimidazolium cations, ^f^ carboxymethyl-dextran, ^g^ polylysine, ^h^ polydiallyldimethylammonium chloride.

## 3. Conclusions

Although prebiotic chemistry experiments using microfluidic devices are few, they have been increasing in recent years. Thanks to the characteristics of microfluidic devices, it is possible that they can be used to simulate different prebiotic environments, such as submarine hydrothermal vents systems in early Earth or rock pores. Additionally, other questions can be approached, as demonstrated by using microfluidic devices to study the chirality problem, compartmentalization phenomena, and catalytic networks that can derive Darwinian evolution. The great potential of microfluidic devices to be used in prebiotic chemistry experiments has yet to be explored. It should be interesting to study more conditions or factors for the experiments that have already been performed. For example, using Y-shaped microfluidic devices, it is possible to study the mineral membrane formation with different physical and chemical conditions such as pH gradient, temperature, chemical composition, or reactant concentrations. In addition, factors such as the presence of relevant organic molecules in mineral membrane formation to evaluate their role as concentrators or catalyzers should be explored. Microdroplet devices that have several applications in the biomedical field also can be used in prebiotic chemistry studies, as shown in the experiments involving reaction networks and compartmentalization. There is great potential to use droplet devices to perform more experiments involving different molecules or conditions. For example, using amphiphilic molecules with prebiotic relevance, to study the probable formation or behavior of what may have been the first membranes. As for studies using microchambers, there is no doubt about their usefulness to study the processes that nucleic acids and some other molecules can undergo in hydrothermal microenvironments. However, it would be also interesting to investigate the processes that other prebiotic molecules, or their precursors, could undergo in those and other kinds of environments.

Perhaps a disadvantage of using microfluidic devices is the individual technical training and laboratory equipment required to manufacturing, operate, and monitor the devices (e.g., manufacture equipment, pumps, and microscopes for observation). However, there are companies dedicated to fabric custom microfluidic devices for diverse purposes. In addition, new methods with low-cost fabrication and materials have already been proposed (e.g., [72,111,112,113,114,115]). The potential use of microfluidic devices constitutes a relatively new approach to prebiotic chemistry, although the present study proves it is rather useful. It would be interesting to use different types of microfluidic devices to respond to key prebiotic chemistry questions. This review pretends to encourage researchers to use microfluidic devices and think about how to use them to perform prebiotic chemistry experiments in novel ways.

## Data Availability

Not applicable.
